# Multiple Myeloma: A Review of Imaging Features and Radiological Techniques

**DOI:** 10.1155/2011/583439

**Published:** 2011-08-08

**Authors:** C. F. Healy, J. G. Murray, S. J. Eustace, J. Madewell, P. J. O'Gorman, P. O'Sullivan

**Affiliations:** ^1^Department of Radiology, Mater Misericordiae University Hospital, Dublin 7, Ireland; ^2^Department of Radiology, University of Texas, MD Anderson Cancer Centre, Houston, TX 77030, USA; ^3^Department of Haematology, Mater Misericordiae University Hospital, Dublin 7, Ireland

## Abstract

The recently updated Durie/Salmon PLUS staging system published in 2006 highlights the many advances that have been made in the imaging of multiple myeloma, a common malignancy of plasma cells. In this article, we shall focus primarily on the more sensitive and specific whole-body imaging techniques, including whole-body computed tomography, whole-body magnetic resonance imaging, and positron emission computed tomography. We shall also discuss new and emerging imaging techniques and future developments in the radiological assessment of multiple myeloma.

## 1. Introduction

Multiple myeloma is a neoplastic disorder of plasma B cells characterised by bone marrow infiltration and overproduction of monoclonal immunoglobulins. It accounts for approximately 10% of all haematological malignancies and 1% of all cancers with an increasing incidence, affecting four in every 100,000 per year [1]. It predominantly affects patients in the seventh decade and has high morbidity and mortality. Patient survival has improved over the past decade with the introduction of novel chemotherapeutic agents [2, 3].

Standard investigations for multiple myeloma includes a complete blood count, serum biochemistry, serum and urine electrophoresis, and the gold standard for diagnosis: bone marrow aspirate and biopsy. The Durie/Salmon staging system introduced in 1975 used skeletal survey as its only radiological criterion [4]. In an effort to standardize treatment approaches and stage the disease as accurately as possible at the time of diagnosis, the Durie/Salmon PLUS staging system has been introduced, integrating the more sensitive imaging techniques of magnetic resonance imaging (MRI), computed tomography (CT), and PET/CT information into its classification system [5].

The role of radiological imaging in multiple myeloma is essentially three fold: in the initial staging of the disease, in the detection and characterisation of complications, and in the evaluation of patient's response to treatment.

## 2. The Biology of Myeloma Bone Disease

 In the past decade, the role of the bone marrow microenvironment has been at the forefront of multiple myeloma research. The “seed and soil” hypothesis was first introduced in the late 1800s by an English surgeon, Dr. Stephen Paget, who proposed a neoplastic growth (the seed, e.g., the myeloma cell) will proliferate in an environment (the soil, e.g., bone marrow environment) that supports its' replication [6]. Since then, in particular in the early 21st century, a multitude of evidence has emerged demonstrating the role of the myeloma cells local environment in augmenting its survival. 

The bony destructive lesions demonstrated by myeloma imaging techniques are caused by myeloma cell-mediated promotion of osteoclast-mediated bony destruction and inhibition of osteoblast-mediated bone anabolism. Myeloma cells attach to osteoclasts directly by numerous adhesion molecules, one example being vascular cell adhesion molecule-1 (VCAM-1), with resultant stimulation of osteoclastogenesis [7].

The effects of myeloma cells on attenuation of osteoblastic activity can be explained, for the most part, by inhibition of osteoblastic differentiation into mature osteoblasts. The main pathway involved in inhibition of osteoblastogenesis is by direct cell-to-cell contact between the mesenchymal stem cells (MSCs) and the myeloma cells. Adhesion of these two entities via VCAM-1 and very late antigen-4 (VLA-4) results in a reduction in Runt-related transcription factor 2 (RUNX2) expression, a critical factor involved in osteoblast transcription [8]. Secondly, myeloma cells secrete factors that inhibit differentiation of osteoblasts, such as Dickkopf 1 (DKK-1), tumour necrosis factor alpha (TNF-*α*), soluble frizzled-related protein-2 (SFRP-2), and Activin A. DKK-1 and SFRP-2 act by inhibiting the Wnt pathway, a pathway that plays a significant role in osteoblastic maturation [9].

## 3. Imaging Techniques

### 3.1. Plain Radiography

**Figure 1:**
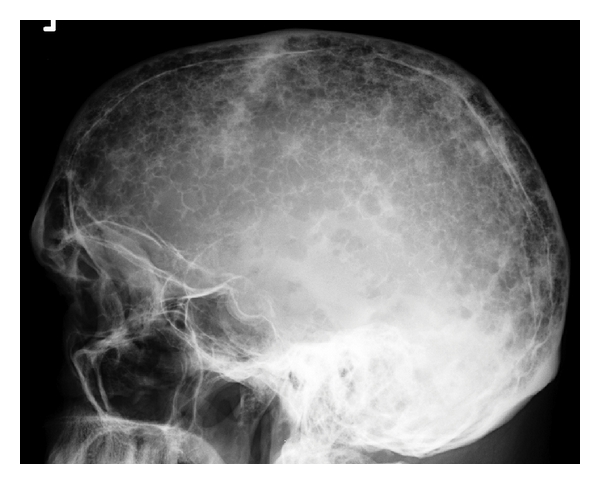
Lateral radiograph skull: diffuse lytic lesions giving classical “pepper pot skull” appearance.

**Figure 2:**
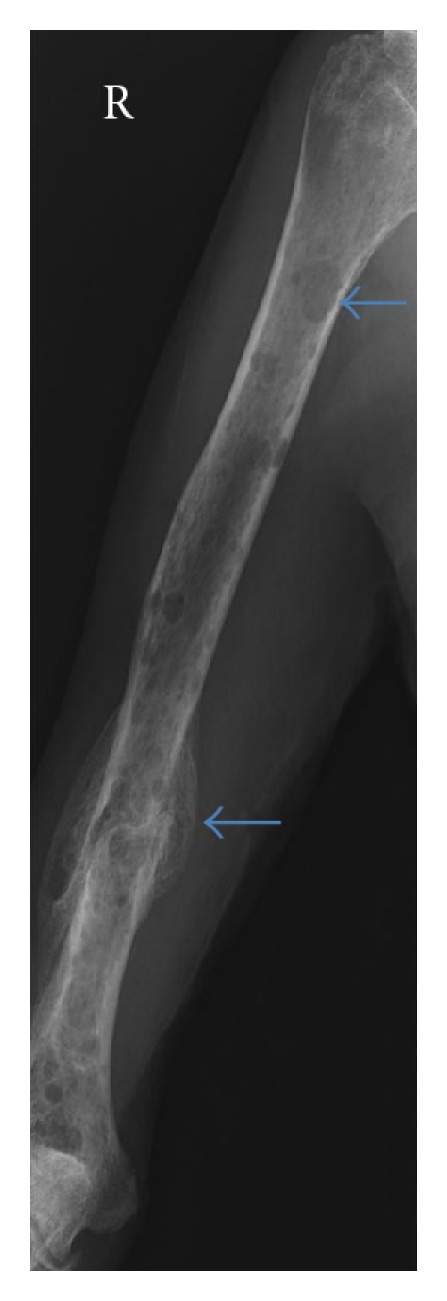
A-P radiograph right humerus: diffuse lytic lesions of the right humerus (arrowed) with old pathological fracture distal diaphysis (arrow).

A full skeletal survey includes a frontal and lateral view of the skull, the cervical, thoracic and lumbar spine, a coned-down frontal view of the dens axis, as well as frontal views of the rib cage, humeri, femora, knees, and pelvis. There is a clear association between the extent of disease, in terms of number of lytic lesions at presentation, and tumour load at diagnosis [4]. Almost 80% of patients with multiple myeloma will have radiological evidence of skeletal involvement on the skeletal survey most commonly effecting the following sites: vertebrae in 66%, ribs in 45%, skull in 40%, shoulder in 40%, pelvis in 30%, and long bones in 25% (Figures 1 and 2) [10]. Plain radiography has the advantage over MRI in detecting cortical bone lesions. It also has the advantage of being universally available, and relatively inexpensive.

One of the major disadvantages of plain radiography is its high false-negative rate of 30–70%, leading to significant underestimation in diagnosis and staging of patients with multiple myeloma [11–13]. Diffuse bone marrow involvement, which may or may not be associated with cortical bone destruction, is not evaluable using conventional radiography. Lytic lesions become apparent on conventional radiography when 30–50% of the bone mineral density is already lost [14, 15]. Furthermore, diffuse osteopenia as a result of multiple myeloma cannot be distinguished on plain radiographs from more common causes of osteopenia, such as senile and postmenopausal osteoporosis [13]. A practical drawback of plain radiography is that varied positions required for radiography films are painful for patients who are often elderly and disabled due to previous pathological fractures.

### 3.2. Computed Tomography (CT)

**Figure 3:**
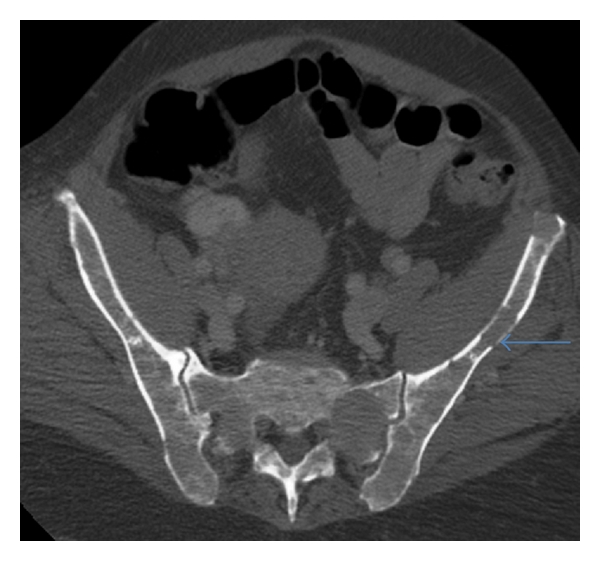
Axial CT pelvis: diffuse myeloma involving the sacrum and iliac bones bilaterally, with cortical destruction of the left iliac bone (arrow).

**Figure 4:**
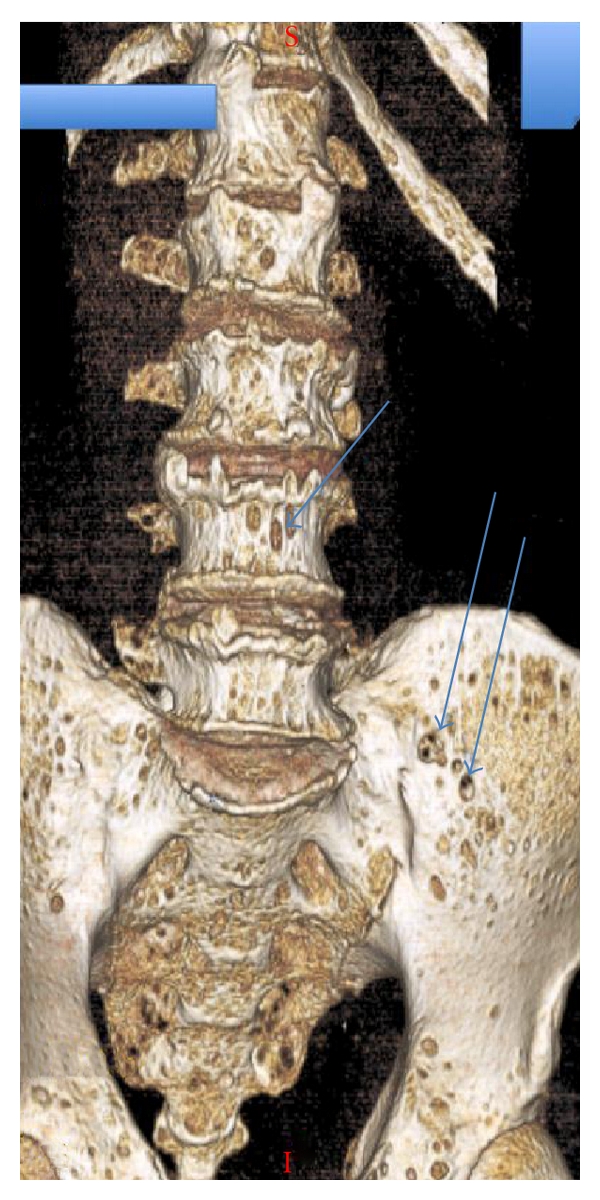
Volume rendering 3-dimensional reconstruction of lumbar spine and pelvis: multiple “punched-out” lytic lesions throughout lumbar spine and pelvis (arrow).

CT is a sensitive imaging modality in detecting the osteolytic effects of multiple myeloma and has a higher sensitivity than plain radiography at detecting small lytic lesions [16]. CT findings in multiple myeloma consist of punched-out lytic lesions, expansile lesions with soft tissue masses, diffuse osteopenia, fractures, and, rarely, osteosclerosis (Figures 3 and 4) [16]. Mahnken et al. compared multidetector row CT with conventional radiography and MR imaging in patients with newly diagnosed multiple myeloma [16]. Multi-detector CT was superior to conventional radiography at defining lytic lesions and, in combination with MR imaging, aided in staging the extent of the disease. CT allowed a more accurate evaluation of areas at risk of fracture than did MR imaging. CT is of use in identifying bone destruction in cases where MR is negative, and hence may provide complementary imaging information. CT has the advantage of accurately demonstrating the presence and extent of extraosseous lesions and is the tool of choice utilised in image guided spinal or pelvic bone biopsy of MR imaging defined focal lesions [17].

**Figure 5:**
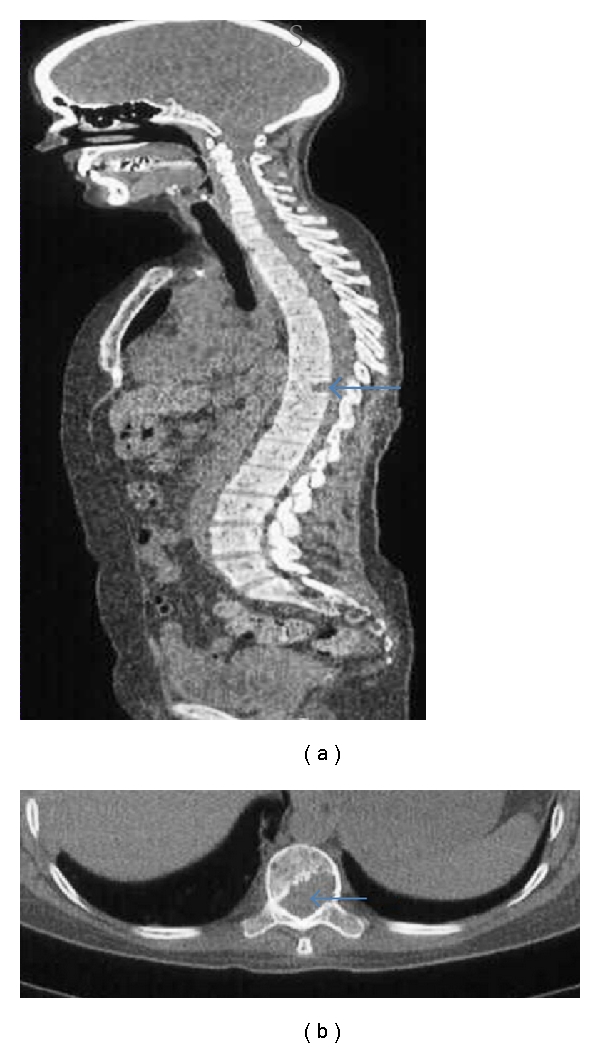
Low dose sagittal whole-body CT (a): note lytic lesion posterior aspect of T10 vertebral body (arrow). Background of extensive osseous permeation from myeloma. Axial CT thorax in the same patient at the level of T10 (b) identifying lytic infiltration of vertebral body (arrow).

Traditionally, whole-body CT has not been used for screening purposes due to the high level of radiation exposure. Low-dose CT techniques are being developed as a possible alternative to plain radiography (Figure 5) [18]. Due to the high intrinsic contrast of bone, the tube current can be lowered significantly (i.e., to 50–100 mA s, depending on the weight of the patient), resulting in an effective equivalent dose in the same range as that of a skeletal survey (4–5 mS v). CT also has the practical advantage of being quick, with the patient lying comfortable on his or her back. Iodine-containing contrast agents, which are contraindicated in patients with multiple myeloma due to the risk of cast nephropathy and renal impairment, are not required for skeletal CT making it an even more attractive screening option. A drawback of CT is that it typically shows persistent bone lesions throughout the course of the disease and, unlike MRI and PET/CT, it cannot assess continued activity of myeloma in areas of prior bone destruction [5].

### 3.3. Whole-Body MRI

**Figure 6:**
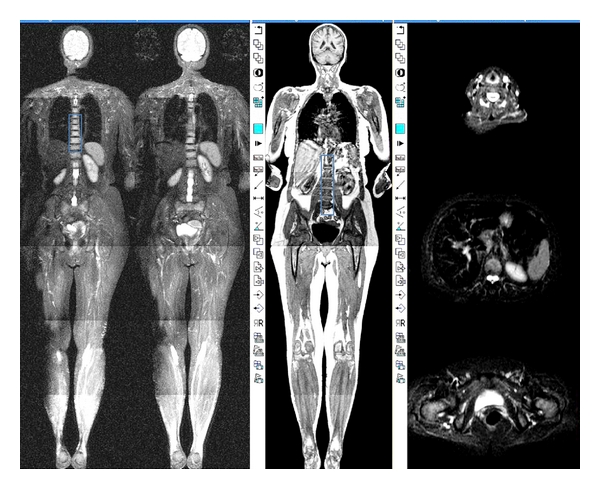
Whole-body MRI coronal and selective axial STIR sequence. Left image: coronal STIR sequence demonstrating T2 bright myelomatous disease throughout the thoracic spine (rectangle). Centre image: coronal T1-weighted sequence demonstrating low signal marrow throughout the lumbar spine due the myelomatous infiltration (rectangle). Right image: 3 axial MRI images at the level of the vocal cords, lumbar spine, and ischium, T1-weighted sequence following administration of contrast.

Whole-body MR (WBMR) has emerged as the most sensitive imaging modality to date at detecting diffuse and focal multiple myeloma in the spine, as well as the extra-axial skeleton [19–21]. Due to its ability to visualise large volumes of bone marrow without inducing radiation exposure and in an acceptable amount of time, MR imaging has become a favoured imaging method for evaluating disease within the bone marrow (Figure 6). MR also has prognostic significance; the number and pattern of lesions detected on MRI correlates very well with treatment outcome and overall survival [5, 20]. The excellent correlation with survival outcomes is the primary reason for inclusion of MRI into the Durie/Salmon PLUS system [5]. It is important to note that MRI predominately reflects marrow infiltration, which may or may not be associated with bone destruction.

The type of MR sequence applied greatly affects the MR images. Multiple sequences have been proposed for use in identifying focal or diffuse disease of the bone marrow. These include spin-echo (T1-weighted and T2-weighted), gradient-echo (T2*-weighted), STIR (short time inversion recovery), and contrast-enhanced spin-echo (with and without fat suppression) sequences [22]. The term “dynamic contrast-enhanced MRI” denotes repeat scanning with high temporal resolution before, during, and after intravenous infusion of a gadolinium-containing contrast agent, using fast T1-weighted sequences [23]. The change in signal intensity over time in a given region is a function of local perfusion, relative blood volume, capillary surface exchange area, vessel permeability, and systemic elimination. Diffusion-weighted imaging (DWI) is increasingly being studied on patients with multiple myeloma [23]. DWI analyses the freedom of movement of interstitial water molecules, which depends on many factors, such as cell density or the presence of organised structures (e.g., fibres). Studies have shown that diffusion is impaired within tumours, and that a decrease in diffusion may indicate disease progression. Effective treatment may cause a transient decrease in diffusion, owing to toxic cell swelling, but thereafter, as the cellular load is reduced, diffusion increases significantly.

**Figure 7:**
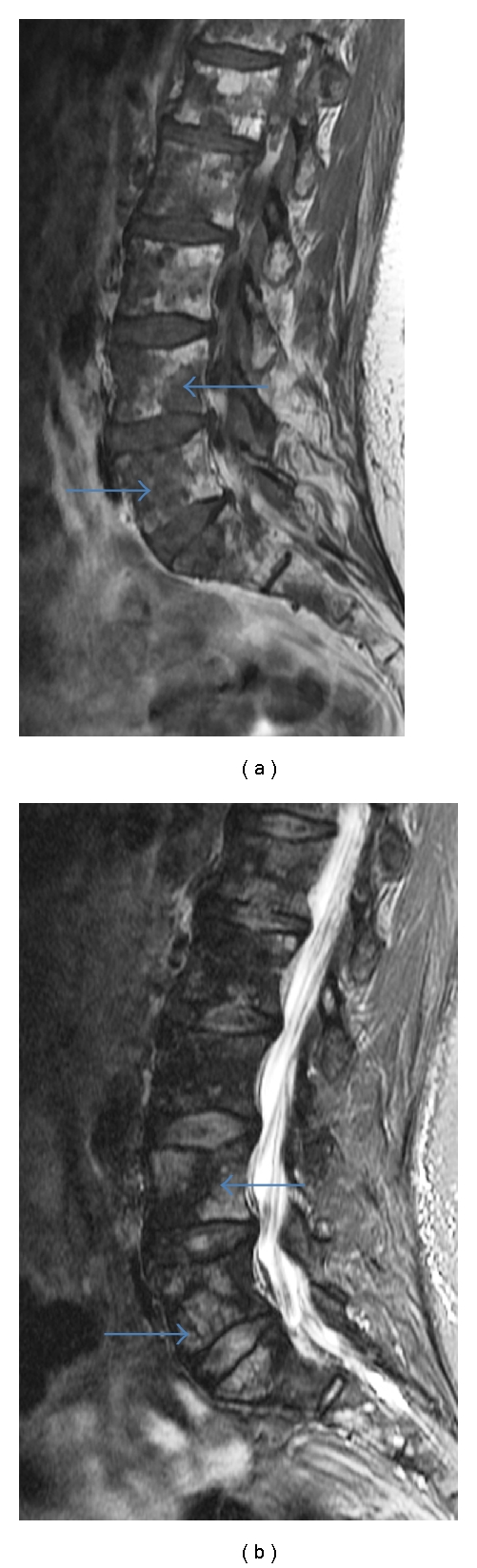
(a) MRI sagittal T1-weighted sequence lumbar spine: diffuse permeative low signal myelomatous marrow lesions throughout the lumbar spine (arrow). (b) MRI sagittal T2-weighted STIR sequence (same patient): diffuse high signal myelomatous marrow lesions throughout the lumbar spine (arrow).

Typical myeloma lesions are marrow based and have low signal intensity on T1-weighted images (Figure 7(a)), and a high signal intensity on T2-weighted sequences and STIR images (Figure 7(b)) and generally show enhancement on gadolinium-enhanced images. Four patterns of marrow involvement have been identified. A normal marrow appearance is present at diagnosis in 50–70% of untreated Durie/Salmon stage 1 and in 20% of untreated Durie/Salmon stage 3 [23]. Other marrow appearances of untreated disease include a focal pattern, a diffuse pattern and a variegated appearance. Thus the main drawback of MR imaging is the lack of specificity. Focal or diffuse patterns may exist at diagnosis and may be a variation of the norm, or reflect an alternative pathological or physiological process. 

Whole-body STIR imaging has gained wider acceptance in detecting occult malignant disease in the skeleton [24, 25]. With ongoing technical advances, such as the moving table, use of multicoil elements, and advances in image processing technology, this technique is becoming more feasible and quicker to perform. Increasing the number of sequences will improve specificity of images, the downside being an increase in acquisition time. These newer techniques enable the inclusion of such sites as the sternum, skull, and ribs, which are usually excluded from standard MR imaging protocols in multiple myeloma. 

**Figure 8:**
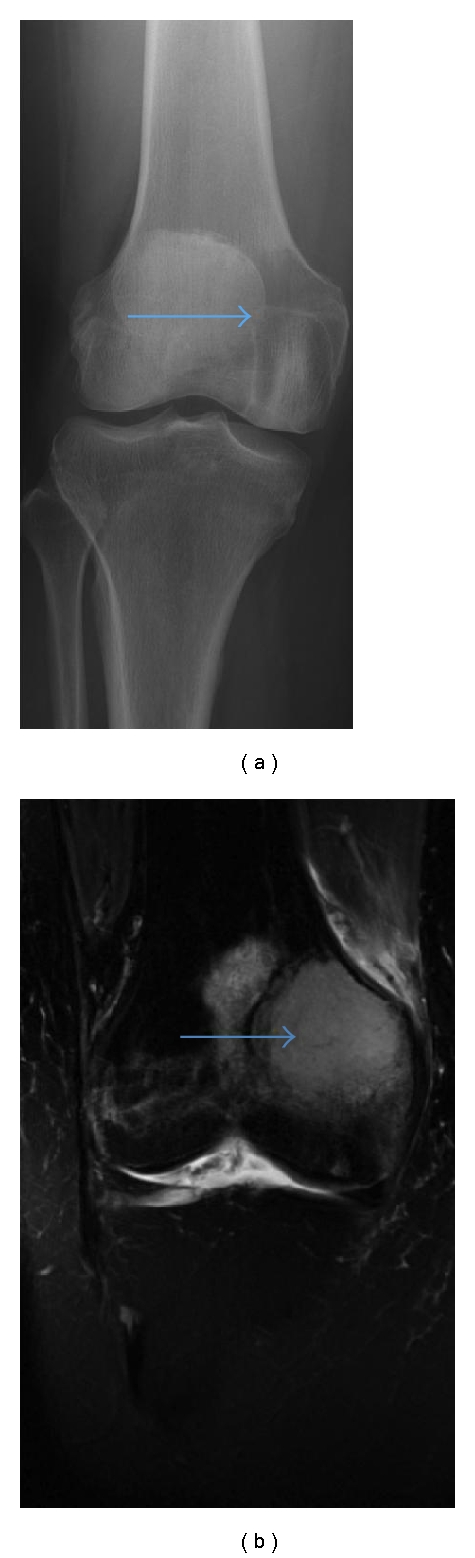
A-P radiograph right knee (a): 4 cm lucency medial femoral condyle (arrow), radiographically difficult to visualise. MRI coronal T2-weighted STIR sequence (b, same patient): high signal 4 cm plasmacytoma medial femoral condyle (arrow).

Dedicated focal MR imaging of the skull, spine, pelvis, or of an extremity is also still a widely utilized technique. MR imaging is routinely required to evaluate the extent and characteristics of a solitary lytic lesion identified at plain radiography (Figure 8). There is often a wide differential for such a lesion, from benign lesions such as osteomyelitis and fibrous dysplasia to malignant processes such as metastases and myeloma.

### 3.4. Nuclear Medicine Imaging (Bone Scintigraphy, PET/CT, and MIBI Scanning) 

#### 3.4.1. Bone Scintigraphy

Bone scintigraphy is of limited use in multiple myeloma. Detection of bone involvement using technetium 99-m (^99m^Tc) labelled diphosphonates relies on the osteoblastic response and activity of the skeletal system for uptake. Multiple myeloma, however, is primarily an osteolytic neoplasm. Lesions that are well defined on isotope bone scans are the result of complications of multiple myeloma, namely, osteoblastic response to a compression fracture of a vertebral body or pelvic insufficiency fracture. Bone scintigraphy may be helpful in evaluating areas not well demonstrated in plain radiography, such as ribs and sternum [26]. In a report comparing the skeletal survey with isotope bone scans, uptake of the radioisotope in radiographically abnormal regions occurred in 44% of cases, normal findings were seen in 48%, and diminished uptake was seen in 8% [27].

#### 3.4.2. PET/CT

Positron emission computed, tomography (PET/CT) is a tomographic nuclear imaging technique that uses a labelled radiopharmaceutical such as ^18^flouro-deoxy-glucose (FDG) injected into the patient, followed by tomographic scanning approximately 10–40 minutes later. Tumour cells can be imaged with this technique due to their high metabolic rate and the resulting high glucose demand, allowing tumour cells to be distinguished from normal cells. Currently, PET/CT is being evaluated in patients with multiple myeloma and may detect early bone marrow involvement in patients with apparently solitary plasmacytoma [28], to assess the extent of active disease, detect extramedullary involvement or evaluate treatment response [29].

**Figure 9:**
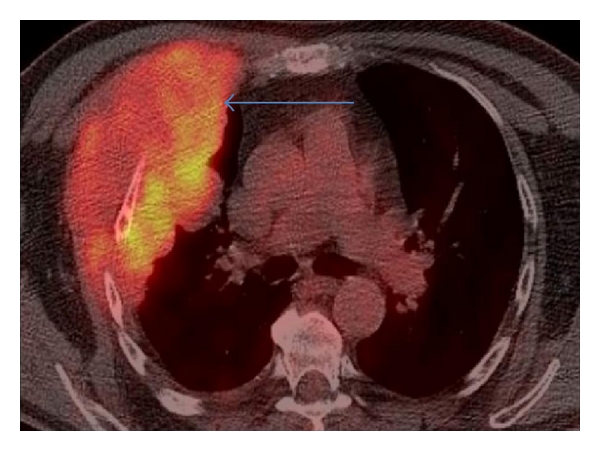
Axial fused PET/CT thorax at the level of the pulmonary bifurcation: massive right-sided chest wall plasmacytoma (arrow).

The main limitation of PET imaging (without the CT component) is limited spatial resolution, which may result in limitations in detecting subcentimetre lytic lesions seen on plain radiography. The advent of fusion scanning combining both the PET component and CT component addresses this issue. In PET/CT fusion scanning, the patient receives an injection of FDG about 1 hour before image acquisition. An initial topogram is acquired to define the range of image acquisition. A spiral CT is then performed. The actual scanning time is shorter for PET/CT (approximately 30 minutes) than for a PET scan alone (approximately 1 hour) as CT data is used to perform attenuation correction (Figure 9).

One of the most significant advantages of PET/CT imaging is its ability to distinguish between active myeloma (FDG positive) and monoclonal gammopathy of undetermined significance (MGUS) or smouldering disease. MGUS is usually negative on PET/CT with neither diffuse marrow uptake nor focal disease in marrow sites [13]. Active myeloma is FDG positive for focal and diffuse abnormities and FDG uptake decreases rapidly after effective therapy. Persistent FDG positivity correlates with early relapse as is discussed later. This rapid response to treatment is converse to that seen with MRI where there may be a time lag of 9–12 months in the reversal of MRI abnormalities despite successful therapy [20].

How does PET/CT compare with other imaging modalities? In a series of 43 patients with multiple myeloma and solitary plasmacytoma, Shirrmeister et al. [28] reported focal tracer uptake on PET/CT scans of 38 of 41 lesions (sensitivity, 93%) with known osteolytic pattern. PET/CT depicted 71 additional lesions missed on plain radiography in 14 patients, which resulted in a change in disease management in 14% of patients studied. Few studies exist directly comparing PET/CT with MRI. A recent study by Shortt et al. [30] compared PET/CT with whole-body MRI in patients with bone biopsy proven multiple myeloma. Whole-body MRI performed better than PET/CT in assessing disease activity having a higher sensitivity (68% versus 59%) and specificity (83% versus 75%) than PET/CT. When used in combination, PET/CT and whole-body MRI were found to have a positive predictive value of 100%. 

False-positive PET/CT scans may arise from inflammatory changes due to active infection, chemotherapy within the preceding 4 weeks, or radiation therapy within the preceding 2-3 months [31].

#### 3.4.3. MIBI Scanning

^99^Technitium sestamibi (MIBI) imaging is an alternative nuclear imaging modality that uses Tc-99m-2-methoxy-isobutyl-isonitrile as a tumour-seeking tracer to identify areas of active disease in a variety of tumours including plasma cell dyscrasias. Using MIBI imaging, it is possible to scan both skeletal lesions and soft tissue lesions and the overall sensitivity is approximately 92% and specificity is 96% [32]. ^99^Tc-MIBI is superior when compared with PET/CT for the visualisation of diffuse disease and despite its limited capacity in detecting focal lesions, this technique may be an alternative option when PET/CT is not available. In comparison to MRI, it has been shown that ^99^Tc-MIBI underestimates the extent of bone marrow infiltration in the spine, especially in patients with low disease stage [33]. ^99^Tc-MIBI may be useful in the detection of indolent disease, as very low-level myeloma is not detectable on PET/CT [5, 34]. An important nuance of ^99^Tc-MIBI scanning is the enhanced uptake of ^99^Tc-MIBI by drug-resistant myeloma cells versus enhanced uptake of FDG by metabolically active myeloma cells [34].

## 4. Imaging of Extramedullary Multiple Myeloma

**Figure 10:**
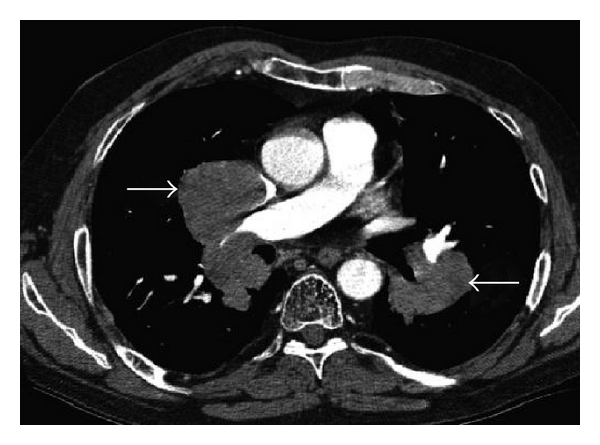
Axial CT thorax postintravenous contrast at the level of T6: diffuse bilateral hilar lymphadenopathy from biopsy-proven multiple myeloma (arrows).

**Figure 11:**
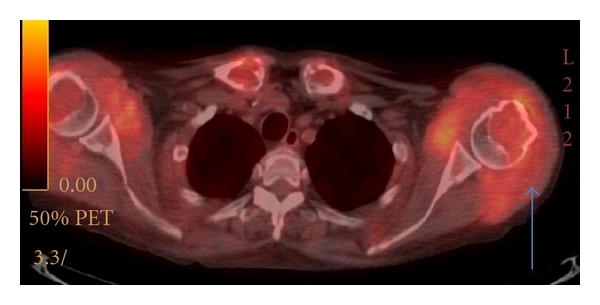
Axial fused PET/CT at the level of T2 vertebra: extensive FDG-avid biopsy-proven amyloid left deltoid muscle in a patient with multiple myeloma (arrow).

Extramedullary multiple myeloma is uncommon. In one review of 432 patients with multiple myeloma, only 19 (4.4%) were identified as having extramedullary multiple myeloma [35]. Multiple myeloma often produces gross sternal expansion and distortion and vertebral body destruction. The pathological tissue appears as soft tissue attenuation similar to muscle at CT, with a degree of enhancement after contrast. MRI typically reveals masses of uniform low signal on T1-weighted image, and uniform high signal on T2-weighted image. Later in the disease cortical breaches may occur, and further local spread from bone may be seen, producing masses in the surrounding soft tissues (Figures 9, 10, and 11). CT is superior to MRI in depicting early cortical breaches [36].

## 5. Imaging of Response to Treatment and Disease Progression

The choice of imaging modality for assessing response to treatment or disease progression generally depends on the findings from the initial workup and the patients' specific treatment regime. Although new or enlarging lesions generally signify disease progression, lytic bone lesions rarely show evidence of healing on plain radiographs, and routine follow-up skeletal survey is of questionable benefit and not routinely indicated in monitoring disease progression or response to treatment. New vertebral compression fractures on plain radiography do not always signify disease progression and may occur even after effective treatment, due to the resolution of the tumour mass that was supporting the bony cortex [37]. 

CT in the followup of treated disease may demonstrate the resolution of extramedullary disease, and the reappearance of a continuous cortical outline. Fatty marrow content may be seen in treated lytic disease in treated cases [38]. As mentioned earlier, one of the nuances of CT is that it cannot assess continued activity of myeloma in areas of prior bone destruction.

Although very accurate, MRI is not ideal for serial monitoring, as it takes 9–12 months for lesions evident on MRI to resolve and be clearly indicative of response [20]. There are a wide spectrum of treatment-induced changes seen on MRI following treatment. MR imaging may fail to demonstrate evidence of regression of myeloma infiltration in the marrow. Focal lesions may shrink or remain unchanged in size. A complete response to treatment may be evidenced by complete resolution of the preceding marrow abnormality, and a partial response demonstrated by conversion of a diffuse to a variegated or focal pattern [22]. A good response to treatment may also be evidenced by a reduction in signal intensity on T2-weighted spin echo images and the absence of contrast-induced rim enhancement that was previously present [19]. 

Diffuse or focal marrow changes may occur following treatment with synthetic growth factors including GCSF and erythropoietin that may simulate active disease on MR or PET/CT imaging [2]. Artificial alterations of FDG activity are typically limited to a 1-month interval after discontinuation of treatment [39].

PET/CT imaging has been shown to be useful in evaluating response to therapy, particularly when other imaging techniques, such as MRI or CT have remained abnormal or inconclusive. PET/CT may identify new sites of disease as well as unsuspected extramedullary spread. Patients appear to have a particularly poor prognosis if abnormal FDG uptake is present following high-dose therapy or stem cell transplantation [40]. In a recent study by Bartel et al. in 2009, it was demonstrated that several imaging parameters related to tumour burden, such as focal lesion number assessed by MRI, and intensity of tumour metabolism on PET/CT, affect survival outcomes [41]. 239 patients with newly diagnosed, untreated symptomatic multiple myeloma enlisted in the Total Therapy 3 program and were evaluated using plain radiographic survey, MRI and PET/CT. Complete FDG suppression in focal lesions and metastatic spread before transplantation conferred superior overall and event-free survival. At 30 months from first autotransplantation, 92% and 89%, respectively, were alive and event-free compared with 71% and 63% among those with less than 100% suppression of FDG uptake in focal lesions or metastatic spread. The presence of more than 3 FDG-avid focal lesions was the leading independent parameter associated with an inferior overall and event-free survival. The prognosis of high-risk patients in this study not achieving complete FDG suppression was poor, which supports the use of serial PET/CT examinations in high-risk patients to individualise therapy and prompt changes to alternative therapies. Importantly, persistent FDG positivity can occur when bone marrow and M-component markers are negative [5].

Despite the plethora of reports on the use of MIBI in the assessment of myeloma patients, the main limiting factor remains its limited sensitivity and specificity, the relatively low numbers of patients included in the reports, and the heterogeneity of clinical situations described [42].

## 6. New Developments

In the late 1990s in the USA, triggered by faster image acquisition, several groups of researchers revisited the earlier work of Dermadian's and Lauturbur's described techniques allowing rapid whole-body imaging using MR imaging. A relatively new whole body coil system is now available with 76 coil elements and 32 high-frequency channels on a 1.5-tesla system. This new system allows for coronal STIR imaging with a field-of-view of 205 cm within 14 minutes with high spatial resolution. 

The first whole-body MR study was performed by Eustace et al. in 1997 [43]. Whole-body MR is now feasible in routine clinical practice. Diffusion weighted imaging of non-CNS tissue has attracted much attention during the past decade. Most experience to date has been gained in differentiating benign from pathological vertebral compression fractures, which can be reliably done when quantitative diffusion measurements are available. Preliminary results exist indicating that this noninvasive technique may be a potential tool for therapy monitoring and for evaluating the effectiveness of modern tumour treatment.

New PET radiopharmaceutical tracers such as 3-flouro-3-deoxy-L-thymidine (^18^F-FLT) which is taken up into cells in relation to the rate of DNA synthesis are being studied [44]. They may visualise the higher cycling activity of haemopoeitic cells in the bone marrow compartment and may be helpful in distinguishing separate haematological disorders.

## 7. Conclusion

It is clear that no one radiological technique in isolation is perfect in accurately staging and monitoring patients with multiple myeloma, and that there are nuances and pitfalls associated with even the most advanced techniques. Whole-body MR, CT, and PET/CT can provide valuable complimentary information when used in the correct setting. As the availability of these techniques increase, so too will their use. This is becoming increasingly important as clinicians strive to best assess the appropriateness and effectiveness of new and changing treatment regimes. Newer and more sensitive imaging techniques, including CT and MR whole-body imaging and functional imaging modalities including PET/CT and ^99^Technitium-2-methoxy-isobutyl-isonitrile open up new avenues in assessing not only morphological disease activity, but also functional disease activity which may be of use in assessing response to treatment as well as tailoring treatment modalities to individual patients.
